# A Survey on the Prevalence of Chlamydia Trachomatis and Mycoplasma Genitalium Infections in Symptomatic and Asymptomatic Men Referring to Urology Clinic of Labbafinejad Hospital, Tehran, Iran

**DOI:** 10.5812/ircmj.8600

**Published:** 2013-04-05

**Authors:** Omid Yeganeh, Mahmood Jeddi-Tehrani, Farhad Yaghmaie, Kourosh Kamali, Hamed Heidari-Vala, Hojjat Zeraati, Nasser Shakhssalim, Saeed Zarei, Leili Chamani-Tabriz

**Affiliations:** 1 Department of Biology, Science and Research Branch, Islamic Azad University, Tehran, IR Iran; 2 Monoclonal Antibody Research Center, Avicenna Research Institute, Academic Center for Education, Culture and Research (ACECR), Tehran, IR Iran; 3 Reproductive Biotechnology Research Center, Avicenna Research Institute, Academic Center for Education, Culture and Research (ACECR), Tehran, IR Iran; 4 Epidemiology and Biostatistics Department, School of Public Health, Tehran University of Medical Sciences, Tehran, IR Iran; 5 Urology and Nephrology Research Center (UNRC), Shahid Labbafinejad Medical Center, Shahid Beheshti University of Medical Science, Tehran, IR Iran; 6 Reproductive Immunology Research Center, Avicenna Research Institute, Academic Center for Education, Culture and Research (ACECR), Tehran, IR Iran

**Keywords:** Asymptomatic Infections, Chlamydia Trachomatis, Infertility, Mycoplasma Genitalium, Prevalence

## Abstract

**Background:**

Chlamydia trachomatis and Mycoplasma genitalium infections are the most prevalent sexually transmitted bacterial infections in the world that cause urogenital infections in both men and women. It appears that infertility is a complication of these infections.

**Objective:**

This study was designed to estimate the prevalence of Chlamydia trachomatis and Mycoplasma genitalium in symptomatic and asymptomatic men and to assess risk factors associated with infection.

**Patients and Methods:**

Urine specimens were collected from 200 men; 100 of them were symptomatic and 100 asymptomatic. Samples were examined by PCR to detect the infections.

**Results:**

C. trachomatis was detected in 20% of symptomatic and in 4% of asymptomatic men (P < 0.001). The prevalence of M. genitalium was revealed to be 12% and 2% in symptomatic and asymptomatic men, respectively (P < 0.01). Four of 100 men in the symptomatic group were infected with both organisms. C. trachomatis infection was associated with dysuria, urethral discharge, testicular swelling, and genital ulcer (P < 0.05). M. genitalium infection was related with dysuria, testis inflammation, pelvic pain and low educational level (P < 0.05). Furthermore, the prevalence of infections at ages 30-39 years was more than other ages.

**Conclusions:**

Considering the role of these bacteria in urogenital infections, a screening test is recommended. Since the PCR assay is a highly sensitive and specific assay for the detection of these bacteria in male urine specimens, it provides a noninvasive technique for routine screening.

## 1. Background

Chlamydia trachomatis and Mycoplasma genitalium are commonly authenticated as urethral pathogens and independently associated with non-gonococcal urethritis (NGU) (1, 2). These infections affect both men and women; about 80% of infected women and 50% of infected men may be asymptomatic (2-4). These bacteria are also sexually transmitted, so it has been suggested that spermatozoa could be as vectors for transmission of these bacteria and cause female genital diseases and infertility (5). Chlamydia trachomatis infections are recognized as the most prevalent sexually transmitted infections among the general population (2, 6-8). Mycoplasma genitalium was first isolated in 1981 from two men with NGU (9). Although M. genitalium had been suggested as a cause of human NGU (10), the accurate role of the Mycoplasma in the etiology of NGU had not been appointed because of the huge difficulty in isolating it from clinical samples (11). It has been detected in the human genitourinary tract, rectum and respiratory tract (12). Partners of infected patients are often also found positive, with a concordance rate similar to that of Chlamydia. Thus, it appears that M. genitalium is the sexually transmitted bacterium and partners should always be looked for and treated to avoid reinfection through the index patient (13). There are not many case histories about the prevalence of C. trachomatis and M. genitalium infections among Iranian men. Regional information is essential to control their spread and help distinguish the effect of these bacteria on the reproductive health of men.

## 2. Objectives

The aim of this study is to investigate simultaneously the prevalence of C. trachomatis and M. genitalium infections in symptomatic and asymptomatic men referring to the urology clinic of Labbafinejad Hospital, Tehran, Iran.

## 3. Patients and Methods

### 3.1. Subjects

One hundred men ranging from 18 to 50 years old with at least one symptom like dysuria, urethral discharge, genital ulcer, pelvic pain, testicular swelling and inflammations of the testicles were selected as symptomatic group and 100 healthy men ranging from 17 to 50 years old with no mentioned symptoms were included as asymptomatic group. Participants delivered a urine sample and filled in a questionnaire containing questions about demographic variables, urogenital symptoms, sexual behavior and social habits. Demographic variables included age, age of first marriage, number of children, educational level, income, and housing status. Sexual behavior included age of first sexual experience, number of permanent sex partners, number of transient partners, and condom usage. The condom usage was divided into always, sometimes, and never usage groups. Furthermore, consumption of cigarette and alcohol were considered as social habits. Included men had not urinated during two last hours (first catch urine) and had not consumed particular antibiotics effect on the C. trachomatis or M. genitalium, like Tetracycline or macrolide group. The study was approved by the Ethics Committee for Medical Research of Avicenna Research Institute, and informed consent was obtained from all participating subjects.

**Figure 1.**
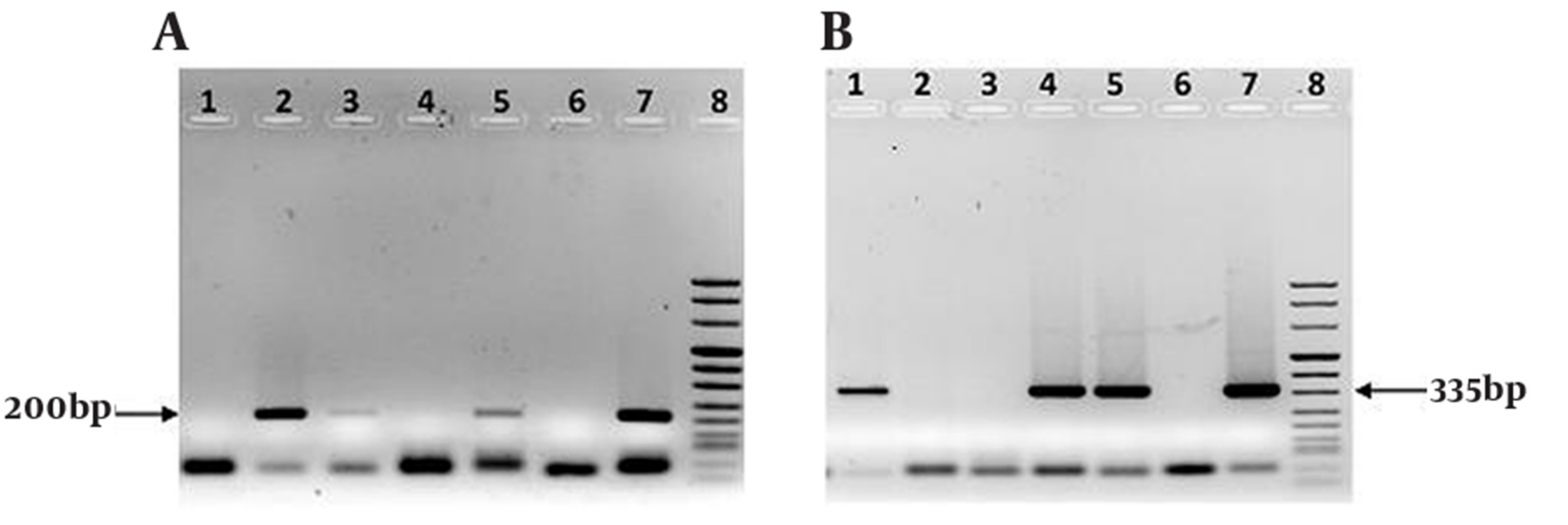
(A) The bond of 200bp indicates the presence of C. trachomatis DNA, Lanes 2, 3, 5: Chlamydia positive patients, Lanes 1, 4: Chlamydia negative men. (B) The bond of 335bp indicates the presence of M.genitalium DNA, Lanes 1, 4, 5: Mycoplasma positive patients, Lanes 2, 3: Mycoplasma negative men. Lane 6: Negative control, Lane 7: Positive control, Lane 8: DNA molecular weight marker VIII in A, B.

Sample collection: A total of 200 male urine specimens were collected from 100 symptomatic and 100 asymptomatic men referring to urology clinic in Labbafinejad Hospital, Tehran, Iran. Up to 50 ml of first-catch urine was collected into sterile containers without preservatives. The specimens were stored at 4°C and transported to the DNA extraction laboratory. DNA extraction and PCR: DNA extraction performed by the method of Sambrook and Russell (14). DNA isolates were dissolved in TE buffer (10 mM Tris-HCl, ph 8.0; 1 mM EDTA). 5 µl of extracted DNA was added to the master mix which consisted of 2.5 µl 10X PCR buffer (without MgCl2) (Roche, Germany), 2mM of MgCl2 (Roche, Germany), 0.4 mM dNTPs, 16 pM of each primer, 1 U of Taq polymerase (Roche, Germany), and distilled H2O to a total volume of 25 µl. The C. trachomatis primers were used to amplify a 200-bp DNA fragment of the orf 8 gene located on cryptic plasmid (15). To detection of M. genitalium, new primers were designed for amplification of a 335bp region of 16s rRNA gene (Table 1). As a positive control of the PCR reaction we used ATCC: VR-902B (LGV II) strain 434 C. trachomatis, and ATCC: 33530 M. genitalium G37. Negative control was prepared by adding 5 µl D.D.W to one tube containing 20 µl of master mix (Roche Molecular Systems). The samples were placed into a programmable thermal cycler (22331, eppendorf, Hamburg, Germany). The amplifications were run under the following program: pre-PCR step 94°C (5 min), so 37 cycles including denaturation at 94°C (30 s) and annealing at 60°C (30 s) and Elongation at 72°C (30 s). At the end, the temperature was held at 72°C for 5 min. The PCR products were detected by band which met the appropriate molecular weight in 1.2% agarose gel stained with ethidium bromide. (Figure 1 A, B).

**Table 1.** Sequence of PCR Primers

Pathogenic Bacterium	Target Gene	Primers	Amplicon Size (bp^a^)
**C. trachomatis**	orf8	F^a^: 5-CTAGGCGTTTGTACTCCGTCA	200
		R^a^: 5-TCCTCAGGAGTTTATGCACT	
**M. genitalium**	16srRNA	F:5- ATAGATACTAGCTGTCGGAGCGAT	335
		R:5-CCAATTTACATTAGCAGTCTCGTTAA	

^a^Abbreviations: F, forward primer; R, reverse primer; bp, base pair

### 3.2. Statistical Analysis

Data were analyzed using descriptive and inferential statistical methods (independent t-test, chi-square test, logistic regression model) in SPSS software (version 13) and P < 0.05 was considered statistically significant. Quantitative values were stated as mean ± standard deviation.

## 4. Results

The ages of 200 men ranged from 17 to 50 years old (mean age, 33.46 ± 8.46; median 34.00). They were evaluated for C. trachomatis and M. genitalium infections by a PCR assay for urine specimens. Symptomatic men (50%) had urethral symptoms (mean age, 33.48 ± 8.4 years), and asymptomatic men (50%) had no urethral symptoms (mean age, 33.44 ± 8.5 years). The prevalence of C. trachomatis (Figure 1A) was 12% overall (20% in symptomatic men and 4% in asymptomatic men, P < 0.001). The prevalence of M. genitalium (Figure 1 B) was 7% overall (12% in symptomatic men and 2% in asymptomatic men, P < 0.01). Only 4 (2%) of 100 men (mean age, 39.75 ± 8.42 years) in the symptomatic group, and none of the asymptomatic men were infected with both organisms. In spite of the highest frequency of C. trachomatis, and M. genitalium urinary tract infections in the men aged 30 to 39 years no significant difference was shown between age groups (Table 2). The prevalence of C. trachomatis and M. genitalium infections in the symptomatic group was about 6 times (95% CI: 1.97 - 18.28) and 6.7 times (95% CI: 1.45 - 30.68) respectively, greater than the asymptomatic group. Multivariate logistic regression model showed significant association between the prevalence of C. trachomatis infection and dysuria, urethral discharge, testicular swelling, and genital ulcer (Table 3). The model also showed significant association between the prevalence of M. genitalium infection and dysuria, pelvic pain, testis inflammation, and low educational level (less than high school) (Table 3). The rates of condom use in all participants were: always, 14.5%; sometimes, 41.3%; and never, 44.2%. The demographic, sexual behavior and social habit characteristics in symptomatic and asymptomatic men are described in (Table 4). There was no statistical difference between two groups of men (P > 0.05). In addition, they did not differ in condom use, income, educational level, and housing status (P > 0.05).

**Table 2.** Effect of Ageand the Prevalence of Chlamydia Trachomatis and Mycoplasma Genitalium Infections Detected by PCR

	C. trachomatis, Positive	M. genitalium, Positive
**Age group, y, No. (%)**		
17-29	6/200 (3)	3/200 (1.5)
30-39	10/200 (5)	7/200 (3.5)
40-50	8/200 (4)	4/200 (2)
**Total, No. (%)**	24/200 (12)	14/200 (7)
**P value**	0.301	0.576

**Table 3.** The statistical AssociationBetween Clinical Symptoms with C. Trachomatis and M. Genitalium Infections

Agent, Clinical symptoms	P value	Odds Ratio	95% CI
**C. trachomatis**			
Dysuria	< 0.001	10.71	2.84 – 40.31
Urethral discharge	0.01	5.57	1.5 – 20.71
Testicular swelling	< 0.05	3.81	1.19 – 12.24
Genital ulcer	< 0.01	6.13	1.93 – 19.44
**M.genitalium**			
Dysuria	< 0.05	5.86	1.37 – 25.07
Pelvic pain	< 0.05	5.5	1.5 – 20.08
Testis inflammation	< 0.05	4.27	1.13 – 16.08
Low educational level	0.01	4.56	1.41 – 14.75

**Table 4.** Demographic, Sexual Behavior and Social Habit Characteristics of Men with and Without Symptoms

Characteristics	Symptomatic men (n = 100)		Asymptomatic men (n = 100)		P value
	Mean ± SD	Median	Mean ± SD	Median	
**Demographic Variables**					
Age, y	33.48 ± 8.41	34.00	33.44 ± 8.54	33.00	0.973
Age of first marriage	24.06 ± 4.30	24.00	24.45 ± 3.45	24.50	0.55
Number of children	1.73 ± 1.30	2.00	1.68 ± 1.33	2.00	0.81
**Sexual behavior**					
Number of permanent sex partners	0.73 ± 0.44	1.00	0.82 ± 0.44	1.00	0.16
Number of transient partners	1.92 ± 0.27	2.00	1.94 ± 0.23	2.00	0.53
Age of first sexual experience	22 ± 4.80	22.00	22 ± 4.85	22.00	0.78
**Social habits, %**					
Cigarette smokers	41		42		0.88
Alcoholics	30		29		0.87

## 5. Discussion

Nowadays, infectious diseases are major preventable health threatening conditions (16, 17). This study investigated the prevalence of C. trachomatis and M. genitalium infections simultaneously, whereas previous studies in Iran mainly assessed the prevalence of single infection. Screening for these infections is important not only to identify infected symptomatic individuals but also to identify asymptomatic infected patients, but routine screening of asymptomatic patients is problematic because of the unwillingness of them to routinely sampling. Considering the role of Chlamydia and Mycoplasma in urogenital infections, NGU, and infertility, it is very apparent that an early diagnosis and proper treatment of these infections can prevent fertility-threatening condition. Urine screening offers an excellent alternative method for detection of these infections (2, 18, 19). It should be noted that there has been less attention in the case of the bacterial causes of male infertility in Iran. However, in some cases, patients are unwilling to participate in clinical trials. Totten et al. (2001) reported the prevalence of M. genitalium was 22% in case patients and 4% in control subjects, and C. trachomatis was detected in 30% of case patients and in 3% of control subjects (1). Wetmore et al. (2011) reported that in men with NGU the prevalence of C. trachomatis and M. genitalium was 22.3% and 12.5%, respectively (20). Shigehara et al. (2011) reported that in Japanese men with urethritis, the prevalence of C. trachomatis and M. genitalium was 26% and 18%, respectively (21). These findings are not inconsistent with the present study and of course the differences in frequency of patients could be related to population studied, patient immunity, the location of study, and the type of specimen (urine vs. urethral discharge).

Compared with our logistic regression model, Leon et al. (2009) reported that chlamydial infection was associated with dysuria in men (22). In addition, Moi et al. (2009) reported that M. genitalium infection was associated with symptoms such as urethral discharge and dysuria (23). Manhas et al. (2009) showed dysuria was more common than urethral discharge in infected men (24). In agreement with the Totten et al. (2001) our findings, showed that patients and control subjects did not differ in sexual behaviors, income, educational level, and cigarette smoking (1). In addition as same as the present study, Baud et al. (2008) reported alcohol, and cigarette were not associated with C. trachomatis infection (25). Klavs and colleagues (2004) using first void urine specimens’ analysis showed that 3.0% of men and 1.6% of women was affected by C. trachomatis infection and regarding to these results, the prevalence was highest in men and women aged 18-24 years (26). Kese et al. (2005) reported the prevalence of C. trachomatis was 19.5% and 10.7% for male and female patients respectively, with the highest prevalence in the group aged 21-30 years in both genders (27). In the present study, the prevalence of C. trachomatis and M. gentalium infections at ages 30-39 years is more than other ages (Table 2). Since non-marital sex is prohibited by Islam's religion, the age trends of STI prevalence in countries such as Iran could be different from other countries. Furthermore, this difference in the age range could be related to the social constraints and cultural considerations (28, 29). This study showed considerable prevalence of these infections in attending patients, which necessitates screening and treatment for the infections. Further investigations are required to determine which target subgroup of the population should be regularly screened, and to evaluate effectiveness of such targeted strategy. Furthermore, with regard to some patients who were infected by both infections, we recommend developing an infectious disease database from patients, at first and then to design a multiplex assay based on obtained prevalence pattern for STD suspected patients profiling.
